# A Phase I Study of KIN-3248, an Irreversible Small-molecule Pan-FGFR Inhibitor, in Patients with Advanced FGFR2/3-driven Solid Tumors

**DOI:** 10.1158/2767-9764.CRC-24-0137

**Published:** 2024-04-30

**Authors:** Benjamin Garmezy, Mitesh J. Borad, Rastilav Bahleda, Cesar A. Perez, Li-Tzong Chen, Shumei Kato, Do-Youn Oh, Paul Severson, Betty Y. Tam, Cheng S. Quah, James J. Harding

**Affiliations:** 1 Sarah Cannon Research Institute, Nashville, Tennessee.; 2 Mayo Clinic Comprehensive Cancer Center, Phoenix, Arizona.; 3 Drug Development Department (DITEP), Gustave Roussy, Villejuif, France.; 4 Sarah Cannon Research Institute at Florida Cancer Specialists, Orlando, Florida.; 5 Kaohsiung Medical University Hospital and Center for Cancer Research, Kaohsiung Medical University, Kaohsiung, Taiwan.; 6 Division of Hematology and Oncology, Department of Medicine, UC San Diego Moores Cancer Center, La Jolla, California.; 7 Seoul National University Hospital, Cancer Research Institute, Seoul National University College of Medicine, Integrated Major in Innovative Medical Science, Seoul National University Graduate School, Seoul, Republic of South Korea.; 8 Kinnate Biopharma, San Francisco, California.; 9 Formerly Kinnate Biopharma, San Francisco, California.; 10 Gastrointestinal Oncology and Early Drug Development Service, Department of Medicine, Memorial Sloan Kettering Cancer Center, New York, New York.

## Abstract

**Purpose:**

Despite efficacy of approved FGFR inhibitors, emergence of polyclonal secondary mutations in the FGFR kinase domain leads to acquired resistance. KIN-3248 is a selective, irreversible, orally bioavailable, small-molecule inhibitor of FGFR1-4 that blocks both primary oncogenic and secondary kinase domain resistance FGFR alterations.

**Experimental Design:**

A first-in-human, phase I study of KIN-3248 was conducted in patients with advanced solid tumors harboring *FGFR2* and/or *FGFR3* gene alterations (NCT05242822). The primary objective was determination of MTD/recommended phase II dose (RP2D). Secondary and exploratory objectives included antitumor activity, pharmacokinetics, pharmacodynamics, and molecular response by circulating tumor DNA (ctDNA) clearance.

**Results:**

Fifty-four patients received doses ranging from 5 to 50 mg orally daily across six cohorts. Intrahepatic cholangiocarcinoma (48.1%), gastric (9.3%), and urothelial (7.4%) were the most common tumors. Tumors harbored *FGFR2* (68.5%) or *FGFR3* (31.5%) alterations—23 (42.6%) received prior FGFR inhibitors. One dose-limiting toxicity (hypersensitivity) occurred in cohort 1 (5 mg). Treatment-related, adverse events included hyperphosphatemia, diarrhea, and stomatitis. The MTD/RP2D was not established. Exposure was dose proportional and concordant with hyperphosphatemia. Five partial responses were observed; 4 in FGFR inhibitor naïve and 1 in FGFR pretreated patients. Pretreatment ctDNA profiling confirmed FGFR2/3 alterations in 63.3% of cases and clearance at cycle 2 associated with radiographic response.

**Conclusion:**

The trial was terminated early for commercial considerations; therefore, RP2D was not established. Preliminary clinical data suggest that KIN-3248 is a safe, oral FGFR1-4 inhibitor with favorable pharmacokinetic parameters, though further dose escalation was required to nominate the MTD/RP2D.

**Significance:**

KIN-3248 was a rationally designed, next generation selective FGFR inhibitor, that was effective in interfering with both FGFR wild-type and mutant signaling. Clinical data indicate that KIN-3248 is safe with a signal of antitumor activity. Translational science support the mechanism of action in that serum phosphate was proportional with exposure, paired biopsies suggested phospho-ERK inhibition (a downstream target of FGFR2/3), and ctDNA clearance may act as a RECIST response surrogate.

## Introduction

Fibroblast growth factor receptors (FGFR), and their ligands, fibroblast growth factors (FGFs) are involved in a wide range of normal developmental and physiologic processes (1–3). To date, over 20 FGFs have been identified, and these mitogens activate four membrane receptor tyrosine kinases (encoded by *FGFR1*, *FGFR2*, *FGFR3*, and *FGFR4*), one receptor that lacks an intracellular domain (encoded by *FGFR5*) as well as a series of coreceptors. Depending upon the cellular and histologic context, ligand-dependent FGFR 1-4 engagement leads to MAPK, PI3K/AKT, and JAK-STAT pathway activation and a cascade of intracellular events required for homeostasis, metabolic, and endocrine function, as well as wound repair.

The FGF/FGFR signaling axis is frequently dysregulated in cancer (2–4). Oncogenesis is mediated by activating genomic alterations in *FGFR* including single-nucleotide variants (SNV), amplification, deletions, fusions, and rearrangements. In addition, autocrine and paracrine FGF secretion in the tumor microenvironment may activate FGFR, drive angiogenesis and epithelial–mesenchymal transition. It is estimated that 10%–30% of all solid tumors are dependent on the FGFR signaling, with 6%–15% of intrahepatic cholangiocarcinoma (ICC) harboring *FGFR2* fusions and arrangements, and 20% of urothelial cancers driven by *FGFR3*-activating mutations or fusions. Actionable genomic alterations are also observed in breast, lung, gastric, and at low frequency in a variety of tumors.

Preclinical evidence indicates that FGFR-driven solid tumors are sensitive to FGFR inhibition. In the clinic, erdafitinib (5–7), a pan-FGFR inhibitor, has demonstrated improved efficacy over cytotoxic chemotherapy in patients with advanced urothelial cancers who progressed on a prior platinum-based regimen. Antitumor activity is also observed for pemigatinib (8), a reversible, ATP competitive, pan-FGFR inhibitor, and futibatinib (9), an irreversible, pan-FGFR inhibitor in patients with FGFR2 fused and rearranged ICC. Finally, tumoral shrinkage has been reported across a range of solid tumors in the context of phase I studies and molecular basket studies (10, 11). Despite the clear efficacy of first-generation pan-FGFR inhibitors, most patients do not attain an objective response, and in those that do, the majority will progress.

Molecular profiling at tumor progression on pan-FGFR inhibitors has identified a series of gatekeeper, molecular brake, and activation loop resistance mutations (12–15). These alterations in *FGFR2* (i.e., N549H/K, V564F, among others) and *FGFR3* (i.e., V555M, N540K, and K650M) have undergone preclinical validation indicating varying degrees of insensitivity to first-generation FGFR inhibitors. KIN-3248 is an orally bioavailable, irreversible, FGFR 1-4 inhibitor that was developed to restore antitumor activity to known resistance mutations, thereby addressing a clear unmet medical need with the broadest potential patient coverage. KIN-3248 exhibited low nanomolar IC_50_ against wild-type FGFR and importantly, common gatekeeper (V564F) and molecular break (N549H) resistance mutations which occur at progression in patients treated with clinically available FGFR inhibitors. Furthermore, KIN-3248 exhibited dose-dependent tumor inhibition in FGFR2/3-driven cholangiocarcinoma, gastric, and bladder cancer xenograft models (16).

On the basis of the preclinical efficacy KIN-3248, we conducted a phase Ib study of KIN-3248 in advanced solid tumor patients harboring *FGFR2* and/or *FGFR3* genomic alterations to test the hypothesis that the agent would exhibit a favorable safety profile with antitumor activity in patients with both FGFR inhibitor naïve and resistant FGFR2/FGFR3-driven solid tumor.

## Materials and Methods

### Study Design

This is a first-in-human, open-label, multicenter, phase Ib study of KIN-3248 in patients with advanced solid tumors harboring oncogenic *FGFR2* and/or *FGFR3* gene alterations (NCT05242822; Supplementary Fig. S1). The study was divided into two parts. The primary objective of Part A was to define the safety, tolerability, and MTD/recommended phase II dose (RP2D) of KIN-3248 using a Bayesian optimal interval (BOIN) design. The primary objective of Part B was to explore KIN-3248 antitumor activity in four expansion cohorts [FGFR2 altered ICC, FGFR2/3 altered urothelial cancer, FGFR2/3 altered treatment-naïve ICC or urothelial cancer, and any solid tumor harboring FGFR2 and/or FGFR3 fusions]. Part B was not completed because of termination of the study by the Sponsor due to commercial concerns. The secondary objectives included antitumor activity in Part A, determination of pharmacokinetics and pharmacodynamics. Exploratory objectives included central confirmation of FGFR2/3 oncogenic driver and nomination of co-occurring genomic alterations by molecular profiling of pretreatment circulating tumor DNA (ctDNA). Tumor-derived ctDNA molecular response kinetics relative to radiographic response was also explored.

The study protocol and its amendments were approved by Institutional Review Boards at each site and the study was conducted in accordance with the standards of Good Clinical Practice and according to the Declaration of Helsinki. All patients provided written informed consent.

### Patient Population

Eligible patients were ≥18 years of age with histologically or cytologically confirmed advanced stage solid tumor. Patients must have received all standard-of-care therapy for their tumor type, or in the opinion of the treating physician, be unlikely to tolerate or to derive clinically meaningful benefit from standard-of-care therapy. *FGFR2* and/or *FGFR3* gene alterations had to be oncogenic or likely oncogenic alterations (Supplementary Table S1) and genotyped from either ctDNA or tumor tissue by a Clinical Laboratory Improvement Amendments–certified laboratory. Patients had to have a RECIST version 1.1 (17) measurable or evaluable disease, maintain an Eastern Cooperative Oncology Group performance state of 0 or 1, and have intact organ function. Prior treatment with FGFR inhibitors was allowed. Key exclusion criteria included: anticancer therapy within 5 half-lives or 28 days of cycle 1 day 1 (C1D1), whichever was shorter, any condition expected to impair oral drug absorption, known clinically active brain metastases, a history and/or current evidence of non–tumor-related alteration of calcium-phosphorous homeostasis, a history and/or current evidence of clinically significant ectopic mineralization/calcification, or a history and/or current evidence of a clinically significant retinal disorder, active cardiovascular condition infection, and pregnancy. A full list of inclusion and exclusion can be found in the Supplementary Materials and Methods.

### Study Treatment

Enrolled patients were assigned a dose of KIN-3248 following BOIN decision rules. Dose level 1 was started at 5 mg oral daily, and patients were escalated on study to 50 mg daily. On the basis of ongoing efficacy, toxicity, and pharmacokinetic analysis, a planned amendment added additional cohorts up to 80 mg orally daily to define the MTD/RP2D. Because of the termination, these additional cohorts were not enrolled. One cycle was 28 days of continuous dosing. Intrapatient dose escalation after safely tolerating two cycles was allowed if per protocol criteria were met. Patients continued therapy until evidence of disease progression, intolerance or unacceptable toxicity, withdrawal of consent, investigator decision, sponsor decision, or death.

### Study Assessments

Patients were evaluated with medical history, physical examinations, and clinical laboratory on days 1, 8, 15, and 22 of cycle 1; days 1 and 15 from cycle 2 through cycle 5; and day 1 of each subsequent cycle. Ophthalmologic examinations were conducted at screening, cycle 2, and then every 4 cycles until treatment termination. Except for hyperphosphatemia, adverse events (AE) were graded according to the NCI-CTCAE (Common Terminology Criteria for Adverse Events) Version 5.0 at each study visit and for approximately 30 days following end of treatment. Hyperphosphatemia was graded according to Supplementary Table S2. Cross-sectional imaging was completed every two cycles and responses were adjudicated locally per RECIST v1.1.

### Pharmacokinetics and Pharmacodynamics

Blood for pharmacokinetic analysis was collected C1D1 (pretreatment and postdose at 0.25, 0.5, 1, 2, 4, 6, 8, 10 hours), C1D2 (pretreatment, postdose at 1 and 2 hours), C1D8 and day 15 (pretreatment, postdose 1 hour), C2D1 (pretreatment, postdose at 0.25, 0.5, 1, 2, 4, 6, 8, 10 hours), and pretreatment and 1 hour postdose on day 1 of C3, C4 and cycle 5. KIN-3248 concentration was determined at each timepoint by an established and validated LC/MS-MS assay.

As FGFR1 is expressed in renal tubules and pharmacologic inhibition of FGFR1 leads to phosphate retention (18), serum phosphate levels were measured at each study visit as a pharmacodynamic biomarker. Paired pretreatment and on-treatment biopsies (cycle 1 day 8 to day 21) were collected and analyzed for phospho-ERK and total ERK by IHC. DUSP6 gene expression was measured in tumor samples using RNA ISH (RNAscope; probes from Advanced Cell Diagnostics).

### Cell-free DNA

Whole blood was collected into Streck Cell Free BCT DNA tubes on cycle 1 day 1 and cycle 2 day 1. Samples were processed to extract plasma followed by cell-free DNA isolation, which was then interrogated by PredicineATLAS, a 600 gene assay able to detect SNVs, indels, rearrangements, fusions, and copy-number gains, as described previously (19).

### Study Endpoint and Biostatistics

The primary objective of Part A dose escalation was to determine the safety and tolerability of oral administration of KIN-3248 including dose-limiting toxicity (DLT) in participants and to identify the MTD and/or the RP2D. The primary endpoints for the overall study, included: the incidence of DLTs, incidence of AEs, treatment-emergent AEs (TEAE), and treatment-related AEs (TRAE). The DLT evaluation period was the first 28 days of cycle 1 and DLT definitions can be found in the Supplementary Materials and Methods. The secondary efficacy endpoints included: objective response rate (ORR), defined as the rate of partial responses (PR) plus complete responses (CR), disease control rate (DCR; CR + PR + stable disease, SD), duration of response, and progression-free survival (PFS). Secondary endpoints included pharamcokinetic parameters of KIN-3248 including maximum observed plasma concentration (C_max_), time to achieve C_max_ (t_max_), and area under the plasma concentration–time curve (AUC) and relationship between exposure (pharmacokinetics) and serum phosphorous levels (pharmacodynamics). When available, paired pretreatment and on-treatment biopsies explored phospho-ERK over total ERK reduction as a tissue-based pharmacodynamic biomarker. The exploratory objective was to describe concordance of genotype by central ctDNA analysis with the genotype nominated locally and to explore ctDNA clearance with RECIST v1.1 response.

The BOIN design used in Part A of the study set a target DLT rate for the MTD of 25%. Safety analysis, including analysis of all AEs, laboratory test values, and vital signs, was conducted on all patients who received at least one dose of KIN-3248. PFS is measured from the date of first KIN-3248 dose until the date of progressive disease or death, whichever is earlier. Pharmacokinetic parameters were calculated from blood plasma concentrations using standard noncompartmental (model-independent) methods.

For each patient with available paired ctDNA, the change in ctDNA after one cycle of treatment was summarized by calculating the mean variant allele frequency (VAF) of all somatic mutations at each timepoint and then taking the ratio as follows: [(mean VAF^C2D1^/mean VAF^C1D1^) −1]*100 (20). For somatic mutations detected in only one sample of a patient-matched pair, the VAF was imputed to be zero for calculation purposes, when the mutation was not detected. Change in match pair ctDNA was correlated both categorically with RECIST v 1.1 response and continuously (Pearson *R*) with the best % change in target lesions. Biomarker analyses were conducted using R [R Core Team 2023 (21)].

### Data Availability

The data generated in this study are available upon request from the corresponding author.

## Results

### Study Status

The study was open at 37 academic and community sites from February 28, 2022 and enrolled a total of 54 patients into six dose cohorts (5 to 50 mg). The first patient enrolled on April 14, 2022, and the last patient enrolled on September 1, 2023. At the time of the data lock of December 12, 2023, 10 patients (18.5%) remained on active treatment. Patients discontinued treatment due to progression of disease (51.9%), study termination by sponsor and adverse events (11.1%, each), and patient withdrawal (7.4%).

### Patient Characteristics

Patient demographics are reported in Table 1. Patients had predominantly ICC (48.1%), gastric (9.3%), urothelial carcinoma (7.4%), and lung cancer (5.6%). Thirty-seven patients (68.5%) had genomic alterations in *FGFR2* including 25 (46.3%) fusions and/or rearrangements, 9 (16.7%) SNVs, 3 (5.6%) fusions plus missense or fusion plus amplification. Seventeen (31.5%) patients harbored *FGFR3* alternations—11 (20.4%) fusions and/or rearrangements, 5 (9.3%) SNVs, and 1 (1.9%) fusion plus amplification. Nine patients have more than one alteration in FGFR2 and/or FGFR3. Patients were treated with a median of 3 (range, 0–6) lines of prior systemic therapy. Twenty-three patients (42.6%) had received prior FGFR inhibitors, of which 17 had ICC.

**TABLE 1:** Demographics

	5 mg *N* = 9	10 mg *N* = 7	20 mg *N* = 8	30 mg *N* = 16	40 mg *N* = 11	50 mg *N* = 3	Overall *N* = 54
Age (years)							
Median	68.0	54.0	64.5	66.5	55.0	58.0	60.5
Sex							
Female	5 (55.6%)	3 (42.9%)	4 (50.0%)	9 (56.3%)	5 (45.5%)	2 (66.7%)	28 (51.9%)
Race							
White	4 (44.4%)	4 (57.1%)	5 (62.5%)	3 (18.8%)	4 (36.4%)	2 (66.7%)	22 (40.7%)
Asian	3 (33.3%)	2 (28.6%)	2 (25.0%)	8 (50.0%)	5 (45.5%)	0	20 (37.0%)
Black or African American	1 (11.1%)	1 (14.3%)	0	1 (6.3%)	0	0	3 (5.6%)
Other/not reported	1 (11.1%)	0	1 (12.5%)	4 (25.0%)	2 (18.2%)	1 (33.3%)	9 (16.7%)
Prior lines of therapy							
0	0	0	0	0	1 (9.1%)	0	1 (1.9%)
1	2 (22.2%)	3 (42.9%)	0	5 (31.3%)	0	0	10 (18.5%)
2 or more	7 (77.8%)	4 (57.1%)	8 (100%)	11 (68.8%)	10 (90.9%)	3 (100%)	43 (79.6%)
Median	2.0	3.0	3.0	3.0	3.0	4.0	3.0
Min, Max	1, 5	1, 6	2, 5	1, 5	0, 4	3, 5	0, 6
Tumor type							
Cholangiocarcinoma	4 (44.4%)	4 (57.1%)	5 (62.5%)	6 (37.5%)	4 (36.4%)	3 (100%)	26 (48.1%)
Gastric, esophageal, and gastroesophageal	0	1 (14.3%)	0	3 (18.8%)	1 (9.1%)	0	5 (9.3%)
Urinary or bladder	1 (11.1%)	0	1 (12.5%)	1 (6.3%)	1 (9.1%)	0	4 (7.4%)
Non–small cell lung cancer	1 (11.1%)	0	0	2 (12.5%)	0	0	3 (5.6%)
Other^a^	3 (33.3%)	2 (28.6%)	2 (25.0%)	4 (25.0%)	5 (45.5%)	0	16 (29.6%)
FGFR2/FGFR3 alterations							
FGFR2	7 (77.8%)	6 (85.7%)	5 (62.5%)	9 (56.3%)	7 (63.6%)	3 (100%)	37 (68.5%)
FGFR3	2 (22.2%)	1 (14.3%)	3 (37.5%)	7 (43.8%)	4 (36.4%)	0	17 (31.5%)
FGFRi prior treatment							
FGFRi: naïve	5 (55.6%)	4 (57.1%)	5 (62.5%)	13 (81.3%)	4 (36.4%)	0	31 (57.4%)
FGFRi: Pretreated	46 (44.4%)	3 (42.9%)	3 (37.5%)	3 (18.8%)	7 (63.6%)	3 (100%)	23 (42.6%)

Abbreviations: FGFGi = FGFR inhibitor; max = maximum; mg = milligrams; min = minimum.

^a^Other = Anal squamous cell carcinoma, brain, breast, cervical, colon, hepatocellular carcinoma, head and neck squamous cell carcinoma, unknown primary, pancreas, neuroendocrine, and salivary.

### Safety and DLT

Of the 54 patients treated in the dose escalation, one DLT occurred in a patient at dose level 1 (5 mg). This patient experienced a treatment-related grade 3 drug hypersensitivity reaction after 8 days characterized by tongue swelling. The event resolved with cessation of therapy and supportive measures. The cohort was expanded to 6 patients with no recurrence at that dose or subsequent higher dose levels. No additional DLTs occurred and the MTD was not reached.

TEAEs of any grade were observed in 53 of 54 patients (98.1%) while TRAEs were observed in 47 of 54 patients (87.0%, Table 2). The most common TRAE of any grade occurring in more than 10% of patients were hyperphosphatemia (64.8%), diarrhea (25.9%), and stomatitis (16.7%).

**TABLE 2:** TEAEs by system organ class and grade

	CTCAE (V5) Grade		
System organ class preferred term	Grade 1–2	Grade 3	Grade 4–5
Patient with any TEAE	28 (51.9%)	18 (33.3%)	7 (13.0%)
Blood and lymphatic system disorders	9 (16.7%)	3 (5.6%)	0
Anemia	10 (18.5%)	2 (3.7%)	0
Eye disorders	12 (22.2%)	0	0
Gastrointestinal disorders	35 (64.8%)	5 (9.3%)	0
Constipation	12 (22.2%)	0	0
Diarrhea	17 (31.5%)	0	0
Dry mouth	8 (14.8%)	0	0
Nausea	6 (11.1%)	0	0
Stomatitis	11 (20.4%)	0	0
General disorders and administration site conditions	18 (33.3%)	0	1 (1.9%)
Fatigue	11 (20.4%)	0	0
Infections and infestations	4 (7.4%)	4 (7.4%)	2 (3.7%)
Investigations	19 (35.2%)	2 (3.7%)	2 (3.7%)
Alanine aminotransferase increased	5 (9.3%)	1 (1.9%)	0
Aspartate aminotransferase increased	4 (7.4%)	2 (3.7%)	0
Blood creatinine increased	7 (13.0%)	0	0
Metabolism and nutrition disorders	30 (55.6%)	10 (18.5%)	0
Decreased appetite	6 (11.1%)	0	0
Hypercalcemia	5 (9.3%)	1 (1.9%)	0
Hyperphosphatemia	29 (53.7%)	8 (14.8%)	0
Musculoskeletal and connective tissue disorders	14 (25.9%)	1 (1.9%)	0
Nervous system disorders	7 (13.0%)	2 (3.7%)	1 (1.9%)
Psychiatric disorders	7 (13.0%)	0	0
Respiratory, thoracic, and mediastinal disorders	7 (13.0%)	1 (1.9%)	1 (1.9%)
Skin and subcutaneous tissue disorders	14 (25.9%)	0	0
Vascular disorders	5 (9.3%)	3 (5.6%)	0

Abbreviations: CTCAE = Common Terminology Criteria for Adverse Events; MedDRA = Medical Dictionary for Regulatory Activities; NCI = National Cancer Institute; TRAE = Treatment-Emergent Adverse Event.

NOTE: *N* represents the number of patients in the Safety Analysis Set in A1 Treatment and is the denominator for percentages. Patients having more than one TRAE within an SOC and PT are counted only once for that SOC/PT at their maximum CTCAE Grade. MedDRA Version 24.0. Grading was determined by Investigator assessment based on NCI-CTCAE, Version 5.0.

TEAE grade ≥3 events occurred in 25 of 54 patients (46.3%) and treatment-related grade 3 events occurred in 11 of 54 patients (20.4%); hyperphosphatasemia was the only event reported in more than 1 patient (14.8%).

Four TEAE deaths occurred—three related to progressive disease and another related to a cardiovascular accident. One death (respiratory failure) was initially coded as treatment related but on detailed clinical review, was considered unrelated.

The median number of cycles was 3 months (range, 1–9). The median duration of treatment was 57 days (range, 8–239). KIN-3248 was interrupted in 16 patients (29.6%) and reduced and discontinued in 7 patients (13.0%) because of TEAEs.

### Pharmacokinetics and Pharmacodynamics

**FIGURE 1:**
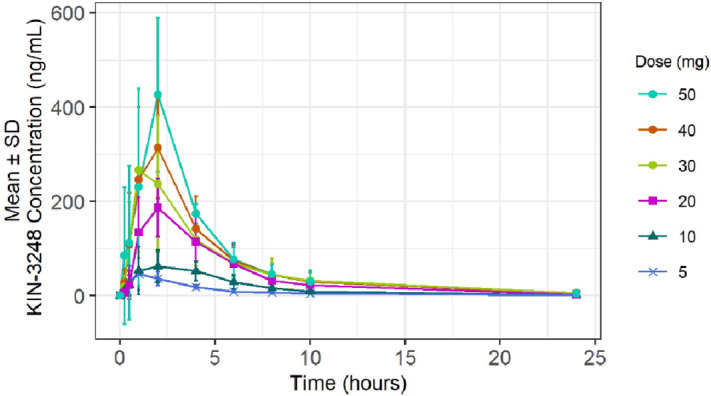
KIN3248 mean concentrations (mean ± SD) in patients following oral daily administration of KIN-3248 during cycle 1 day 1 and day 2.

KIN-3248 concentration over time following treatment on cycle 1 day 1 is shown in Fig. 1. The maximum plasma concentration typically occurred 2 to 4 hours after dosing and exhibited monophasic decay with steady-state geometric mean half-life (t_1/2_) of approximately 4 hours, which was not dose dependent (Supplementary Table S3). Similar apparent clearance (CL/F) and volume of distribution (V_z_/F) values were observed across doses with CL/F ranging from 23,300 to 32,100 mL/hour and V/F ranging from 135,000 to 200,000 mL. Exposure was generally dose proportional with dose. Accumulation was not observed in the first two cycles. In exploratory analysis (Supplementary Fig. S2), there was not a qualitative difference between Asian (China, Taiwan, Korean) and non-Asian patients. Dose exposure modeling was used to estimate the therapeutic dose of KIN-3248. The lower bound was set as a dose equivalent of 20 mg futibatinib and the upper bound was based on targeting exposure (unbound AUC) at 80% tumor growth inhibition in the mouse xenograft models, that was estimated at approximately 60 mg.

**FIGURE 2:**
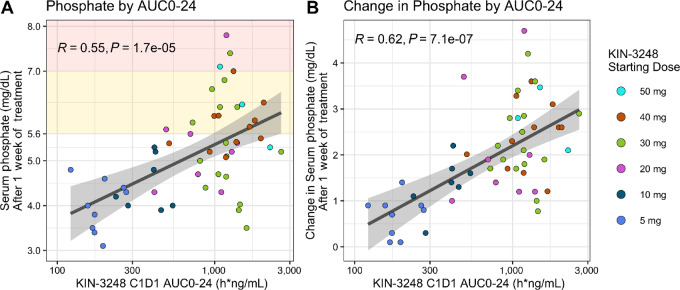
Serum phosphate by KIN-3248 C1D1 exposure. Baseline and on-treatment serum phosphate were measured locally in all 54 participants. **A,** Scatter plot of serum phosphate (mg/dL) after approximately 1 week on KIN-3248 by KIN-3248 C1D1 exposure (AUC0–24). Significant correlation between KIN-3248 exposure and serum phosphate level after approximately 1 week of treatment (nominal C1D8: actual study days 6, 7, 8 or 9). Linear fit with SE. Pearson *R* = 0.55, *P* = 1.7e-5. **B,** Scatter plot of initial change in serum phosphate by KIN-3248 C1D1 exposure (AUC0–24). Significant correlation between C1D1 KIN-3248 exposure and the initial change (∼1 week) in serum phosphate (PhosC1D8 – PhosC1D1). Pearson *R* = 0.62, *P* = 7.1e-7. One participant was missing the C1D1 phosphate measurement, so the screening measurement was used as the baseline value for that participant.

Serum phosphate increased with dose and exposure of KIN-3248 as seen in Fig. 2 and Supplementary Fig. S3. Eleven paired biopsy samples were available for IHC and there was a trend toward reduction in phosho-ERK relative to total-ERK (Supplementary Fig. S4).

### Antitumor Activity

Of 54 patients enrolled, 5 achieved a PR and none achieved a CR with an of ORR 9.25%. Response occurred in dose levels 20–40 mg and were seen in FGFR2/3 fusion patients [5 patients (20%)] and included 1 patient with pancreatic cancer with FGFR2-SEPT7 fusion, 1 patient with breast cancer with FGFR2-ABLIM1 fusion plus an N549D mutation (molecular brake), 1 patient with cancer of unknown primary with FGFR2-ERC1 fusion, and 2 patients with gastroesophageal junction cancer with FGFR2 fusions. Of 31 who were FGFR inhibitor naïve, there were 4 responders of which 3 are confirmed. Of 23 patients who were FGFR inhibitor experienced, there was 1 confirmed responder. Twenty patients (37.0%) achieved SD. The DCR was 46% in the total population. Six patients had progressive disease as best response or were not evaluable.

### Exploratory ctDNA Analysis

**FIGURE 3:**
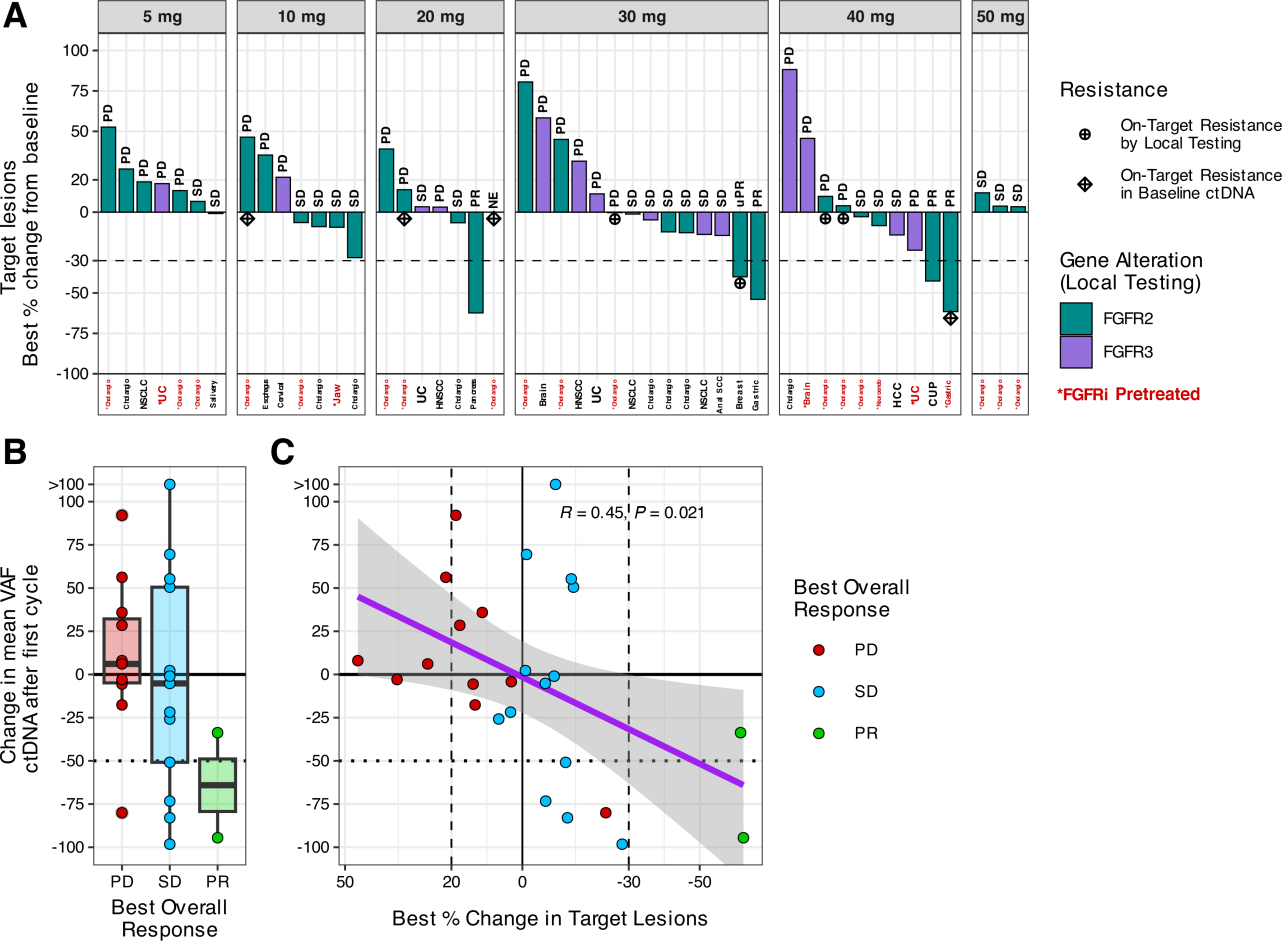
Antitumor activity. **A,** Best percent change in target lesions by KIN-3248 starting dose level. Bars are color-coded by the gene (FGFR2 or FGFR3) that was altered according to the molecular report provided for study eligibility evaluation (local testing). Bars are labeled with the best overall response for each evaluable participant. *X*-axis is labeled with the tumor type of each participant, with the labels color-coded (black or red) to show which participants have been previously treated (*) with one or more FGFR inhibitors. Participants with documented on-target acquired resistance mutations, either by local testing or central ctDNA analysis, are indicated with distinct symbols. **B,** Twenty-six patients were evaluable for efficacy and were tested by central ctDNA analysis. Box and whisker plot of change in mean variant allele frequency of ctDNA after first cycle of KIN-3248 treatment by best overall response. **C,** Scatter plot of change in mean variant allele frequency of ctDNA after first cycle of KIN-3248 treatment by best % change in target lesions. X-axis direction is reversed. Linear fit with SE. Pearson *R* = 0.45, *P* = 0.021.

Of 54 patients, 30 patients underwent central molecular profiling of pretreatment cell free DNA. Of 30 patients tested, 29 had tumor-derived ctDNA detected but only 19 (63%) had confirmation of *FGFR2/3* status as documented by local genotyping—12 had fusions and 7 had missense mutations (Supplementary Fig. S5). In one case, local (*FGFR3* Y373C) and central testing (*FGFR3* S249C and EPB41L2-FGFR2) were discordant. The most frequent co-occurring alterations were in *TP53 (59%)*, *BAP1 (24%),* and *PIK3CA (17%)*. Oncogenic *KRAS*, *NF1/2*, *MET* mutations or amplifications were found in 6 patients. Of the 23 patients who had received prior FGFR inhibitors, 10 patients were tested and acquired resistance mutations in FGFR were detected in 4 (Fig. 3A; Supplementary Table S4). Of these patients, 1 patient with gastric cancer responded to study treatment. Among 6 patients with pretreated ICC tested by central ctDNA analysis, 3 (50%) were found to have FGFR2 kinase domain resistance mutations. The best response in these 3 patients was progressive disease. Of the 29 patients with detectable ctDNA baseline, we had 29 cycle 2 day 1 samples to explore the kinetics of ctDNA clearance and its association with RECIST version 1.1 response. Twenty-six patients were evaluable for both RECIST response and ctDNA response. Six patients had greater than a 50% decrease in the mean VAF after 1 cycle of KIN-3248 treatment. There was a trend toward greater reductions in ctDNA in patients with better overall response (Fig. 3B) and decreases in target lesions (Fig. 3C).

## Discussion

Prior studies indicate that acquired resistance to first-generation FGFR inhibitors is mediated, in part, by point mutations in the kinase domain of FGFR2/3 driven cancers. KIN-3248 is a rationally designed, orally bioavailable, next-generation, pan-FGFR 1-4 inhibitor that retains antitumor efficacy despite acquisition of these genomic alterations in preclinical models. Given these data, we conducted a first-in-human phase I study of KIN-3248 in patients with FGFR2 and/or FGFR3-driven solid tumors. KIN-3248 was safe and tolerable with a favorable pharmacokinetic profile. Pharmacodynamic analysis, from blood as assessed by serum phosphorous and preliminary analysis of paired biopsies, supported KIN-3248 target engagement. A signal of antitumor activity was also observed in both FGFR inhibitor naïve and experienced solid tumors. Importantly, application of serial ctDNA sampling and its real-time analyses suggested that early reduction in circulating tumor (i.e., molecular response) may associate with RECIST response. The totality of our data suggested that the MTD/RP2D was indeed higher, and although we had planned to amend the study to explore higher dose levels the clinical trial was terminated. Unfortunately, we were not able to define the MTD or nominate a RP2D. Despite failure of the study to meet its primary objective, a meaningful proportion of patients were treated with available liquid-based pharmacokinetic, pharmacodynamic, and ctDNA correlatives, thus enabling useful scientific information that may aid in the development of effective next-generation FGFR2/3 inhibitors.

Common AEs observed on this study included dermatologic, gastrointestinal, and ocular toxicity, as well as hyperphosphatemia—which is consistent with previous observations related to the safety of pan-FGFR inhibitors. Importantly, there were no treatment-related deaths and the rates of dose reduction or study termination due to a TRAE were relatively low and in line with other clinically available FGFR inhibitors. Ocular toxicity, which was frequent, and typically low grade, was reversible in all cases (22). We also observed a variety of dermatologic AEs, that were mostly grade 1 or 2 events, and manageable with established protocols (23). Nail disorders and onychomadesis were observed as grade 1 AEs within the dose levels tested and duration of treatment. Hyperphosphatemia, a direct on-target toxicity due to FGFR1 in renal tubule, was observed in a dose-dependent and exposure-dependent fashion. Importantly, phosphate binders mitigated this toxicity allowing for continued dose escalation. It remains unclear whether hyperphosphatemia would become a liability at doses above 50 mg daily. Acknowledging the hazards of cross-trial comparison, the toxicity data for KIN-3248 are similar to those that are clinical available and taken together help to credential KIN-3248 as a clinical grade pan FGFR inhibitor (5–11).

Notably, KIN-3248 exhibited linear exposure relative to dose and did not accumulate with repeat dosing. The global design of the study was a relative strength of the study, allowing for the potential exploration of the impact on differential pharmacogenomic and ethnicity on drug exposure. Exposure was relatively similar across populations. Although serum phosphate levels were used as a serologic pharmacodynamic biomarker, the initial design of the study also called for paired biopsies to assess DUSP6 and p-ERK, downstream mediators of FGFR signaling (24). Because of the study termination, these analyses were only completed in a small number of patients. Although phosho-ERK relative to total ERK appeared to decrease in paired biopsy, multiple factors including variable sample collection relative to dose, tumor heterogeneity, complexity of FGFR downstream signaling, as well as small sample size, limit a firm conclusion on tissue-based pharmacodynamic of KIN-3248. Despite these limitations, it is notable that 50% of samples tested had a reduction in p-ERK, which supports the proposed mechanism of action. Notably, exposure-efficacy modeling indicated that efficacious doses should range between 30 and 60 mg orally daily.

In line with our pharmacokinetic/pharmacodynamic modeling, tumor shrinkage and objective responses were observed at 20 mg orally and above. Responses were observed in a histologic agnostic manner, and in both FGFR naïve and experienced population. Of note, a patient with RAS wild-type FGFR2 fused pancreatic cancer attained a deep and durable response, and it is now established that such alterations are seen in <1% patients with pancreatic ductal adenocarcinoma, indicating the need to identify these patients with clinical grade genotyping. Unfortunately, due to termination of the study, exploration of higher dose levels and expansion of cohorts at 60 mg and above was not possible. Additional data at this level and above would be required to make firm conclusion regarding both safety and the antitumor activity of KIN-3248.

A critical clinical question, is whether KIN-3248, based on its novel design, would allow continued activity in an FGFR2/3 resistance population. Another question, based on the cellular potencies, as described by Tyhonas and colleagues, is whether a higher range of exposure could potentially afford more effective coverage against secondary acquired resistance mutations (16). It is also now clear, that innate resistance may, in part, be related to reactivation of ERK signaling (25). In line with these data, a recent retrospective analysis of serial cell-free DNA of 17 patients receiving FGFR inhibition found that 52% of patients developed mutations in the MAPK pathway, that might contribute to acquired FGFR inhibitor resistance and that a combination of FGFR and MEK inhibitors block RAF-MEK-ERK reactivation and resistance mutations *in vitro* (26). In 29 patients with available ctDNA, oncogenic *KRAS*, *NF1/2*, *MET* mutations or amplifications were found in 6 patients. More work is required to define the clinical importance of these preliminary observations.

Finally, exploratory utilization of ctDNA confirms several prior findings. Namely that ctDNA analysis must be used in complementary fashion to tumor-based assays as approximately 40% of mutations and fusion were not detected at central confirmation (27). These false negatives likely reflect assay sensitivity, especially for structural variants, as well as factors specific to tumor, such as shed rate and disease burden (28, 29). Analyses also provide some of the first data that ctDNA clearance in FGFR2/3-driven tumors is associated with RECIST response. These data are hypothesis generating given the *ad hoc* nature of the design and small size but provide needed preliminary evidence to evaluate ctDNA clearance prospectively as both a surrogate of tumor response and survival. The initial design of the study also called for both tumor and liquid biopsy at progression of disease to nominate novel genomic mechanisms of resistance to KIN-3248, though all patients with response were on going at the time of the data lock.

The limitation of the study was its early closure—leading to short follow up time, missing data related to paired biopsies and ctDNA correlates, and an inability to establish the RP2D necessary to explore antitumor activity in both histologic specific and agnostic cohorts as well as in an FGFR inhibitor resistant population. In summary, KIN-3248 was safe and tolerable with favorable pharmacokinetics with a signal of antitumor activity but clinical development was terminated. Other selective isoform FGFR2 and FGFR3 inhibitors continue in their clinical development and these studies may clarify the optimal strategies for drug the FGF/FGFR axis in cancer [NCT05544552, NCT04526106, NCT05614739 (30)].

## Supplementary Material

Supplementary Methods Inclusion Criteria, Exclusion Criteria, Dose Limiting Toxicity Criteria for First Cycle

Supplementary Figure 1 Supplemental Figure 1 - Study Schema

Supplementary Figure 2 Supplemental Figure 2 - Mean KIN-3248 Concentrations over time for Asian versus non-Asian Patients

Supplementary Figure 3 Supplemental Figure 3 - Serum Phosphate Over Time by KIN-3248 Dose Level

Supplementary Figure 4 Supplemental Figure 4 - Pharmacodynamic effect of KIN-3248 treatment was evaluated in serial sections of paired biopsy samples by measuring changes in phospho-ERK and DUSP6 gene expression.

Supplementary Figure 5 Supplemental Figure 5 - Somatic Mutation Profiles in Baseline ctDNA

Supplementary Table 1 Supplemental Table 1 - Pathogenic / Likely-pathogenic FGFR2 Gene Alterations

Supplementary Table 2 Supplemental Table 2 - Management of Hyperphosphatemia Observed with FGFR Inhibitors

Supplementary Table 3 Supplemental Table 3 - Summary of Pharmacokinetic Parameters of KIN-3248 (Cycle 1 Day 1)

Supplementary Table 4 Supplemental Table 4 - FGFRi pre-treated patients with kinase domain resistance mutations identified in centrally tested ctDNA

## References

[1] Xie Y , Su N, Yang J, Tan Q, Huang S, Jin M, . FGF/FGFR signaling in health and disease. Signal Transduct Target Ther 2020;5:181. 32879300 10.1038/s41392-020-00222-7 PMC7468161

[2] Krook MA , Reeser JW, Ernst G, Barker H, Wilberding M, Li G, . Fibroblast growth factor receptors in cancer: genetic alterations, diagnostics, therapeutic targets and mechanisms of resistance. Br J Cancer 2021;124:880–92. 33268819 10.1038/s41416-020-01157-0 PMC7921129

[3] Babina IS , Turner NC. Advances and challenges in targeting FGFR signalling in cancer. Nat Rev Cancer 2017;17:318–32. 28303906 10.1038/nrc.2017.8

[4] Touat M , Ileana E, Postel-Vinay S, Andre F, Soria JC. Targeting FGFR signaling in cancer. Clin Cancer Res 2015;21:2684–94. 26078430 10.1158/1078-0432.CCR-14-2329

[5] Loriot Y , Necchi A, Park SH, Garcia-Donas J, Huddart R, Burgess E, . Erdafitinib in locally advanced or metastatic urothelial carcinoma. N Engl J Med 2019;381:338–48. 31340094 10.1056/NEJMoa1817323

[6] Loriot Y , Matsubara N, Park SH, Huddart RA, Burgess EF, Houede N, . Erdafitinib or chemotherapy in advanced or metastatic urothelial carcinoma. N Engl J Med 2023;389:1961–71. 37870920 10.1056/NEJMoa2308849

[7] Siefker-Radtke AO , Necchi A, Park SH, Garcia-Donas J, Huddart RA, Burgess EF, . Efficacy and safety of erdafitinib in patients with locally advanced or metastatic urothelial carcinoma: long-term follow-up of a phase 2 study. Lancet Oncol 2022;23:248–58. 35030333 10.1016/S1470-2045(21)00660-4

[8] Abou-Alfa GK , Sahai V, Hollebecque A, Vaccaro G, Melisi D, Al-Rajabi R, . Pemigatinib for previously treated, locally advanced or metastatic cholangiocarcinoma: a multicentre, open-label, phase 2 study. Lancet Oncol 2020;21:671–84. 32203698 10.1016/S1470-2045(20)30109-1 PMC8461541

[9] Goyal L , Meric-Bernstam F, Hollebecque A, Valle JW, Morizane C, Karasic TB, . Futibatinib for FGFR2-rearranged intrahepatic cholangiocarcinoma. N Engl J Med 2023;388:228–39. 36652354 10.1056/NEJMoa2206834

[10] Meric-Bernstam F , Bahleda R, Hierro C, Sanson M, Bridgewater J, Arkenau HT, . Futibatinib, an irreversible FGFR1–4 inhibitor, in patients with advanced solid tumors harboring FGF/FGFR aberrations: a phase I dose-expansion study. Cancer Discov 2022;12:402–15. 34551969 10.1158/2159-8290.CD-21-0697 PMC9762334

[11] Pant S , Schuler M, Iyer G, Witt O, Doi T, Qin S, . Erdafitinib in patients with advanced solid tumours with FGFR alterations (RAGNAR): an international, single-arm, phase 2 study. Lancet Oncol 2023;24:925–35. 37541273 10.1016/S1470-2045(23)00275-9 PMC11224843

[12] Goyal L , Shi L, Liu LY, de la Cruz FF, Lennerz JK, Raghavan S, . TAS-120 overcomes resistance to ATP-competitive FGFR inhibitors in patients with FGFR2 fusion-positive intrahepatic cholangiocarcinoma. Cancer Discov 2019;9:1064–79. 31109923 10.1158/2159-8290.CD-19-0182 PMC6677584

[13] Goyal L , Saha SK, Liu LY, Siravegna G, Leshchiner I, Ahronian LG, . Polyclonal secondary FGFR2 mutations drive acquired resistance to FGFR inhibition in patients with FGFR2 fusion-positive cholangiocarcinoma. Cancer Discov 2017;7:252–63. 28034880 10.1158/2159-8290.CD-16-1000 PMC5433349

[14] Silverman IM , Hollebecque A, Friboulet L, Owens S, Newton RC, Zhen H, . Clinicogenomic analysis of FGFR2-rearranged cholangiocarcinoma identifies correlates of response and mechanisms of resistance to pemigatinib. Cancer Discov 2021;11:326–39. 33218975 10.1158/2159-8290.CD-20-0766

[15] Varghese AM , Patel J, Janjigian YY, Meng F, Selcuklu SD, Iyer G, . Noninvasive detection of polyclonal acquired resistance to FGFR inhibition in patients with cholangiocarcinoma harboring FGFR2 alterations. JCO Precis Oncol 2021;5:PO.20.00178. 34250419 10.1200/PO.20.00178 PMC8232836

[16] Tyhonas JS , Arnold LD, Cox JM, Franovic A, Gardiner E, Grandinetti K, . Discovery of KIN-3248, an irreversible, next generation FGFR inhibitor for the treatment of advanced tumors harboring FGFR2 and/or FGFR3 gene alterations. J Med Chem 2024;67:1734–46. 38267212 10.1021/acs.jmedchem.3c01819

[17] Eisenhauer EA , Therasse P, Bogaerts J, Schwartz LH, Sargent D, Ford R, . New response evaluation criteria in solid tumours: revised RECIST guideline (version 1.1). Eur J Cancer 2009;45:228–47. 19097774 10.1016/j.ejca.2008.10.026

[18] Erben RG , Andrukhova O. FGF23-Klotho signaling axis in the kidney. Bone 2017;100:62–8. 27622885 10.1016/j.bone.2016.09.010

[19] Renouf DJ , Loree JM, Knox JJ, Topham JT, Kavan P, Jonker D, . The CCTG PA.7 phase II trial of gemcitabine and nab-paclitaxel with or without durvalumab and tremelimumab as initial therapy in metastatic pancreatic ductal adenocarcinoma. Nat Commun 2022;13:5020. 36028483 10.1038/s41467-022-32591-8 PMC9418247

[20] Zhang Q , Luo J, Wu S, Si H, Gao C, Xu W, . Prognostic and predictive impact of circulating tumor DNA in patients with advanced cancers treated with immune checkpoint blockade. Cancer Discov 2020;10:1842–53. 32816849 10.1158/2159-8290.CD-20-0047 PMC8358981

[21] R Core Team (2023). R: a language and environment for statistical computing. R foundation for statistical computing, Vienna. Available from: https://www.R-project.org/.

[22] Francis JH , Harding JJ, Schram AM, Canestraro J, Haggag-Lindgren D, Heinemann M, . Clinical and morphologic characteristics of fibroblast growth factor receptor inhibitor-associated retinopathy. JAMA Ophthalmol 2021;139:1126–30. 34473206 10.1001/jamaophthalmol.2021.3331 PMC8414363

[23] Lacouture ME , Sibaud V, Anadkat MJ, Kaffenberger B, Leventhal J, Guindon K, . Dermatologic adverse events associated with selective fibroblast growth factor receptor inhibitors: overview, prevention, and management guidelines. Oncologist 2021;26:e316–26. 33021006 10.1002/onco.13552 PMC7873330

[24] Nakanishi Y , Mizuno H, Sase H, Fujii T, Sakata K, Akiyama N, . ERK signal suppression and sensitivity to CH5183284/Debio 1347, a selective FGFR inhibitor. Mol Cancer Ther 2015;14:2831–9. 26438159 10.1158/1535-7163.MCT-15-0497

[25] Wu Q , Zhen Y, Shi L, Vu P, Greninger P, Adil R, . EGFR inhibition potentiates FGFR inhibitor therapy and overcomes resistance in FGFR2 fusion-positive cholangiocarcinoma. Cancer Discov 2022;12:1378–95. 35420673 10.1158/2159-8290.CD-21-1168 PMC9064956

[26] DiPeri TP , Zhao M, Evans KW, Varadarajan K, Moss T, Scott S, . Convergent MAPK pathway alterations mediate acquired resistance to FGFR inhibitors in FGFR2 fusion-positive cholangiocarcinoma. J Hepatol 2024;80:322–34. 37972659 10.1016/j.jhep.2023.10.041 PMC11900356

[27] Brannon AR , Jayakumaran G, Diosdado M, Patel J, Razumova A, Hu Y, . Enhanced specificity of clinical high-sensitivity tumor mutation profiling in cell-free DNA via paired normal sequencing using MSK-ACCESS. Nat Commun 2021;12:3770. 34145282 10.1038/s41467-021-24109-5 PMC8213710

[28] Supplee JG , Milan MSD, Lim LP, Potts KT, Sholl LM, Oxnard GR, . Sensitivity of next-generation sequencing assays detecting oncogenic fusions in plasma cell-free DNA. Lung Cancer 2019;134:96–9. 31320002 10.1016/j.lungcan.2019.06.004

[29] Mc Connell L , Gazdova J, Beck K, Srivastava S, Harewood L, Stewart JP, . Detection of structural variants in circulating cell-free DNA from sarcoma patients using next generation sequencing. Cancers 2020;12:3627. 33287361 10.3390/cancers12123627 PMC7761870

[30] Subbiah V , Sahai V, Maglic D, Bruderek K, Toure BB, Zhao S, . RLY-4008, the first highly selective FGFR2 inhibitor with activity across FGFR2 alterations and resistance mutations. Cancer Discov 2023;13:2012–31. 37270847 10.1158/2159-8290.CD-23-0475 PMC10481131

