# Supplementary material for: A Phase I Study of KIN-3248, an Irreversible Small-molecule Pan-FGFR Inhibitor, in Patients with Advanced FGFR2/3-driven Solid Tumors
Source: Cancer Res Commun. 2024 Apr 30;4(4):1165–73. doi: 10.1158/2767-9764.CRC-24-0137 (PMC11060137; doi:10.1158/2767-9764.CRC-24-0137)
Supplement: Supplementary Methods — Inclusion Criteria, Exclusion Criteria, Dose Limiting Toxicity Criteria for First Cycle [file crc-24-0137-s01.pdf]

Supplemental Methods

Inclusion Criteria

1. Participant has provided written informed consent prior to initiation of any study-specific procedures.
2. Men or women at least 18 years of age (or ≥ age of majority in local jurisdictions) at the time of signing the informed consent form (ICF).
3. Histologically or cytologically confirmed diagnosis of advanced-stage malignancy, as follows:
    - Part A - Any type of advanced tumors harboring FGFR2 and/or FGFR3 gene alterations
    - Part B Cohort 1 - Advanced ICC with FGFR2 gene alterations who previously demonstrated clinical benefit to FGFRi treatment and discontinued due to disease progression
    - Part B Cohort 2 - Advanced UC with FGFR2 and/or FGFR3 gene alterations who previously demonstrated clinical benefit to FGFRi treatment and discontinued due to disease progression
    - Part B Cohort 3 - Advanced ICC or UC with FGFR2 and/or FGFR3 gene alterations who are FGFRi naïve
    - Part B Cohort 4 - Advanced solid tumors (other than ICC or UC) with FGFR2 and/or FGFR3 gene alterations who are FGFRi naïve or were previously treated with FGFRi
4. Participants must have either received prior standard of care therapy (including FGFR-related agents approved in local jurisdictions) appropriate for their tumor type and stage of disease or, in the opinion of the Investigator, be unlikely to tolerate or to derive clinically meaningful benefit from standard of care therapy (applicable to Parts A & B).

   In Part B Cohorts 1 and 2, participants with ICC or UC driven by FGFR2 and/or FGFR3 gene alterations should have previously received an FGFR inhibitor, approved by the local regulatory agency or as an investigational agent within a clinical trial where such treatment exists (hereafter referred to as "such treatment"). Additionally, such treatment must have resulted in clinical benefit, for example objective tumor shrinkage (i.e., complete response [CR] or partial response [PR]) or sustained disease control (i.e., continuous stable disease for at least 8 weeks) prior to confirmed progressive disease. In Cohorts 3 and 4, FGFRi naïve participants must have received at least one line of prior systemic therapy. In Cohort 4, other solid tumors previously treated with FGFRi do not need to have demonstrated clinical benefit to such treatment.
5. Participant's tumor has documented FGFR2 and/or FGFR3 alteration in blood and/or tumor per local assessment as detected by DNA sequencing using a comprehensive next-generation sequencing assay (e.g., FoundationOne® CDx or Guardant360® CDx), break-apart fluorescence in situ hybridization (FISH), or other validated assay. Potential participants harboring an amplification as the only FGFR2 or 3 alteration would not be considered eligible.

   Genomic analysis of tumor tissue or circulating tumor-derived (blood) nucleic acids (ctDNA) must have been conducted in a Clinical Laboratory Improvement Amendments (CLIA)-

certified laboratory (in US) or in accordance with local regulatory requirements (in other countries).

6. Willing to provide an archived tumor tissue specimen (formalin-fixed paraffin embedded [FFPE] specimen) obtained within the last 5 years, if available.
7. Willing to undergo pre-treatment tumor biopsy, if medically feasible. The availability of a FFPE tumor biopsy specimen obtained within 2 months prior to consent from participants who have not received intervening systemic anti-cancer therapy will satisfy the pretreatment biopsy requirement.
8. Measurable or evaluable disease by Response Evaluation Criteria in Solid Tumors (RECIST) v1.1. For Part B, measurable disease only.
9. Eastern Cooperative Oncology Group (ECOG) Performance Status of 0 or 1.
10. An estimated life expectancy of at least 3 months in the opinion of the Investigator.
11. Adequate hematological lab assessments at screening, as follows:
    - Absolute neutrophil count (ANC) ≥ 1000/mm^3 (1.0 x 10^9 /L)
    - Platelet count ≥ 75,000/ mm^3 (75x 10^9 /L)
    - Hemoglobin ≥ 9.0 g/dL (at least 7 days since most recent blood transfusion)
12. Adequate renal laboratory assessments, as follows:
    - Estimated creatinine clearance ≥ 50 mL/min as calculated using the Cockcroft-Gault method or a method standard for the institution
13. Adequate hepatic laboratory assessments, as follows:
    - ALT and aspartate transaminase ≤ 3 x upper limit of normal (ULN), or ≤ 5 x ULN in the presence of liver metastases.
    - Serum total bilirubin ≤ 1.5 x ULN (< 3.0 x ULN for participants with documented Gilbert's syndrome).
    - ALP ≤2.5 x ULN (≤ 5 x ULN in case of bone metastasis, biliary tract involvement or liver metastases).
14. Adequate chemistry laboratory assessments, as follows:
    - Sodium ≥ 130 mEq/L or within institutional normal limits.
    - Potassium ≥ 3.6 mmol/L or within institutional normal limits.
    - Phosphate ≤ 1.5 x ULN
15. Able to swallow, retain, and absorb oral medications.

Exclusion Criteria

1. Known clinically active or progressive brain metastases. Participants who have had brain metastases resected or have received radiation therapy ending at least 4 weeks prior for whole brain radiation or 2 weeks prior for gamma knife, stereotactic radiation, or other localized radiotherapy to Cycle 1 Day 1 are eligible if they meet all of the following criteria:
    - Residual neurological symptoms Grade ≤ 2

- On stable doses of dexamethasone (i.e., no increase in dose for preceding 14 days), if applicable AND
- No new lesions in the brain appearing on most recent magnetic resonance imaging (MRI)

2. History and/or current evidence of any of the following disorders:
   - Non-tumor related alteration of calcium-phosphorous homeostasis that is considered clinically significant in the opinion of the investigator
   - Ectopic mineralization/calcification, including but not limited to soft tissue, kidney, intestine, or myocardia and lung, considered clinically significant in the opinion of the investigator.
   - Corneal or retinal disorder/keratopathy including, but not limited to, bullous/band keratopathy, corneal abrasion, inflammation/ulceration, and keratoconjunctivitis and considered clinically significant in the opinion of the investigator.
3. Participants with any other active malignancy within 3 years prior to enrollment, except for adequately treated basal cell or squamous cell skin cancer, or carcinoma in situ of the cervix, Bowen's disease. Participants with prostate intraepithelial neoplasm and ≤ 6 Gleason grade prostate cancer may also be considered. Participants with a history of other curatively treated cancers must be reviewed by the Sponsor prior to entering the study.
4. Baseline standard 12 lead ECG that demonstrates clinically relevant abnormalities that may affect participant safety or interpretation of study results (e.g., baseline QTcF > 450 ms (calculated using Fridericia's formula), complete left bundle branch block, signs of an acute or indeterminate age myocardial infarction, ST T interval changes suggestive of active myocardial ischemia, second- or third-degree atrioventricular block, or serious bradyarrhythmias or tachyarrhythmias).
5. Myocardial infarction within 6 months of Cycle 1 Day 1, symptomatic congestive heart failure (New York Heart Association > Class II), unstable angina, or cardiac arrhythmia requiring medication.

   Exception: Participants who have atrial fibrillation with heart rate controlled by medication are eligible to enroll.
6. GI tract disease causing the inability to take oral medication, malabsorption syndrome, requirement for intravenous alimentation, uncontrolled inflammatory GI disease (e.g., Crohn's disease, ulcerative colitis).
7. Active, uncontrolled bacterial, fungal, or viral infection, including (but not limited to) hepatitis B virus (HBV), hepatitis C virus (HCV), and known human immunodeficiency virus (HIV) or acquired immune deficiency syndrome (AIDS)-related illness. The following circumstances apply:
   a. Coronavirus disease (COVID)-19/severe acute respiratory syndrome corona virus 2 (SARS-CoV2): This protocol excludes participants with active infections, as noted above. While SARS-CoV2 testing is not mandated for entry into this protocol, testing should follow local clinical practice standards. If a participant has a positive test result for SARS-CoV2 infection, is known to have

asymptomatic infection or is suspected of having SARS-CoV2, he/she is excluded.

b. HIV: Seropositivity can be confirmed per local regulations, however the following considerations apply in equivocal cases:
    i. Participants whose viral load is negative or HIV serology is negative may be eligible upon discussion with Sponsor.
    ii. HIV seropositive participants who are otherwise healthy and at low risk for AIDS-related outcomes could be considered eligible. Potential eligibility for a specific HIV-positive protocol candidate should be evaluated and discussed with the Sponsor prior to any screening. HIV infected participants must be on anti-retroviral therapy (ART) and have wellcontrolled HIV infection/disease defined as:
        - Participants on ART must have a CD4+ T cell count > 350 cells/mm3 at time of screening
        - Participants on ART must have achieved and maintained a viral suppression defined as confirmed HIV RNA level below 50 copies/mL or the lower limit of qualification (below the limit of detection) using the available assay at the time of screening and for at least 12 weeks prior to screening
        - Participants on ART must have been on a stable regimen, without changes in drugs or dose modification, for at least 4 weeks prior to study entry (Day 1)
    iii. The potential for DDIs will be taken into consideration.
    iv. For participants in China and Japan, those who are confirmed HIV antibody positive are excluded.

c. HBV/HCV: Relevant laboratory tests should be performed at Screening. Refer to Centers for Disease Control website: (https://www.cdc.gov/hepatitis/index.htm) for further details.

d. HBV: Participants who are Hepatitis B surface antigen positive are eligible if they have received HBV anti-viral therapy for at least 4 weeks and have undetectable HBV viral load prior to enrollment. Participants should remain on anti-viral therapy throughout study intervention. For participants in China, Japan, and Taiwan, when either hepatitis B surface antibody or hepatitis B core antibody positive, the HBV DNA must be < 200 IU/ml or 1000 copies/mL to be eligible under this criterion.

e. HCV: Participants with a history of HCV infection are eligible if HCV viral load is undetectable at screening and have completed curative anti-viral therapy at least 4 weeks prior to enrollment.

8. Unresolved acute toxicities from prior anti-tumor therapy, defined as not having resolved to CTCAE ≤ 1, to baseline severity, or to levels otherwise dictated in the eligibility criteria (alopecia and ≤Grade 2 peripheral sensory neuropathy are not considered an exclusion criterion).

9. Anti-cancer therapy (chemotherapy, antibody therapy, molecular targeted therapy, retinoid therapy, hormonal therapy or investigational agent) within 5 half-lives or 28 days of Cycle 1 Day 1, whichever is shorter.

   Exception: concurrent use of hormone deprivation therapy for hormone-refractory prostate cancer or breast cancer is permitted.
10. Currently enrolled in another interventional investigational drug study,
11. Use of strong inhibitors or inducers of CYP3A, including grapefruit/grapefruit related citrus fruits (e.g., Seville oranges and pomelos) and any herbal medicine (e.g., St. John's wort) within 1 week of Cycle 1 Day 1 and planned use during the study is prohibited.
12. Participants sustaining major surgery (defined as a complex procedure performed under regional or general anesthesia) with a recovery period of at least 3 weeks prior to study enrollment.
13. Blood pressure ≥ 150/100 mmHg that cannot be controlled despite optimal medical therapy.
14. Men and women of reproductive potential who are unwilling to practice acceptable methods of effective birth control while on study through 30 days (180 days for woman of childbearing potential [WOCBP] in France and South Korea) or 90 days (men) after receiving the last dose of study drug. Acceptable highly effective contraception is defined as sexual abstinence (refraining from heterosexual intercourse; men, women), vasectomy, or tubal ligation or a combination of a+b or a+c or b+c):
    a. Use of oral, injected or implanted hormonal methods of contraception or other forms of hormonal contraception that have comparable efficacy (failure rate <1%)
    b. Intrauterine device (IUD) or intrauterine system (IUS)
    c. Barrier methods of contraception: Condom or Occlusive cap (diaphragm or cervical/vault caps) with spermicidal foam/gel/film/cream/vaginal suppository.

    A WOCBP is a female who 1) has achieved menarche at some point 2) has not undergone a hysterectomy or bilateral oophorectomy or 3) has not been naturally postmenopausal (amenorrhea following cancer therapy does not rule out childbearing potential) for at least 12 consecutive months (has had at least menses at any time preceding 12 consecutive months).
15. Women who are lactating/breast feeding or who plan to breastfeed while on study through 1 week after receiving the last dose of study drug.
16. Women with a positive pregnancy test or planning to become pregnant while on study through 1 week after receiving the last dose of study drug.
17. Participant has known sensitivity to any of the products to be administered during dosing.
18. Participant will not be available for protocol-required study visits or procedures, to the best of the participant and Investigator's knowledge.

Dose Limiting Toxicity Criteria for First Cycle (28 Days)

Feature	Criteria
**Hematologic**	• Febrile neutropenia- any grade<br>• Grade 4 neutropenia > 5 days<br>• Grade ≥ 3 neutropenia with infection<br>• Grade 4 thrombocytopenia<br>• Grade ≥ 3 thrombocytopenia with bleeding event<br>• Grade 4 anemia
**Non-Hematologic**	• Grade ≥ 3 nausea for 3 days or more despite optimal medical support
	• Grade 3 vomiting or diarrhea that persist for > 24 hours despite anti-emetic, anti-diarrheal medication<br>• Persistent Grade 3 increased AST or persistent Grade 3 increased ALT)<br>• Presence of liver metastases: AST or ALT > 8x ULN or AST/ALT > 5 ULN<br>• Laboratory abnormalities that satisfy Hy's Law (i.e., ALT or AST elevation > 3X ULN, total bilirubin elevation > 2X ULN, absence of initial findings of cholestasis or absence of elevation of alkaline phosphatase to > 2 X ULN), and no other reason can be found to explain the combination of increased ALT/AST and total bilirubin<br>• Grade 3 fatigue for ≥ 7 days<br>• Grade 3 electrolyte abnormality regardless of duration; except for isolated grade 3 laboratory abnormalities not associated with clinical sequelae and managed with supplementation/appropriate management within 72 hours of their onset<br>• Any other Grade ≥ 3 AEs including laboratory investigations *
**Other**	• Participant's death that is not clearly attributable to underlying disease or extraneous cause<br>• Any treatment-related AE that is assessed by the Investigator and Sponsor as a DLT<br>• Any clinically important or persistent treatment related toxicities that led to significant delay of drug administration for > 14 days
