# Supplementary figures and images for: A Phase I Study of KIN-3248, an Irreversible Small-molecule Pan-FGFR Inhibitor, in Patients with Advanced FGFR2/3-driven Solid Tumors
Source: Cancer Res Commun. 2024 Apr 30;4(4):1165–73. doi: 10.1158/2767-9764.CRC-24-0137 (PMC11060137; doi:10.1158/2767-9764.CRC-24-0137)

Supplemental Figure 1: Study Schema

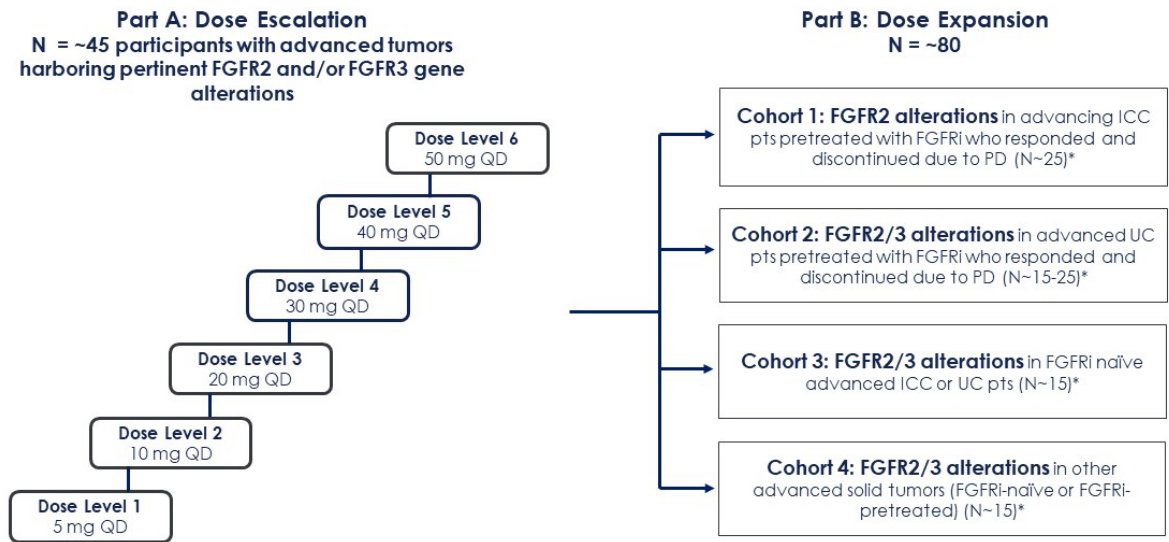

Supplement: Supplementary Figure 1 — Supplemental Figure 1 - Study Schema [file crc-24-0137-s02.pdf]
