# Supplementary material for: A Phase I Study of KIN-3248, an Irreversible Small-molecule Pan-FGFR Inhibitor, in Patients with Advanced FGFR2/3-driven Solid Tumors
Source: Cancer Res Commun. 2024 Apr 30;4(4):1165–73. doi: 10.1158/2767-9764.CRC-24-0137 (PMC11060137; doi:10.1158/2767-9764.CRC-24-0137)

Supplemental Figure 2: Mean KIN-3248 Concentrations over time for Asian versus non-Asian Patients. Mean (SD) plasma KIN-3248 concentration versus time profiles stratified by Asia vs. non-Asia Preliminary data shows PK was generally similar between Asian (China, Taiwan, Korea) versus Western (North America, EU) participants.

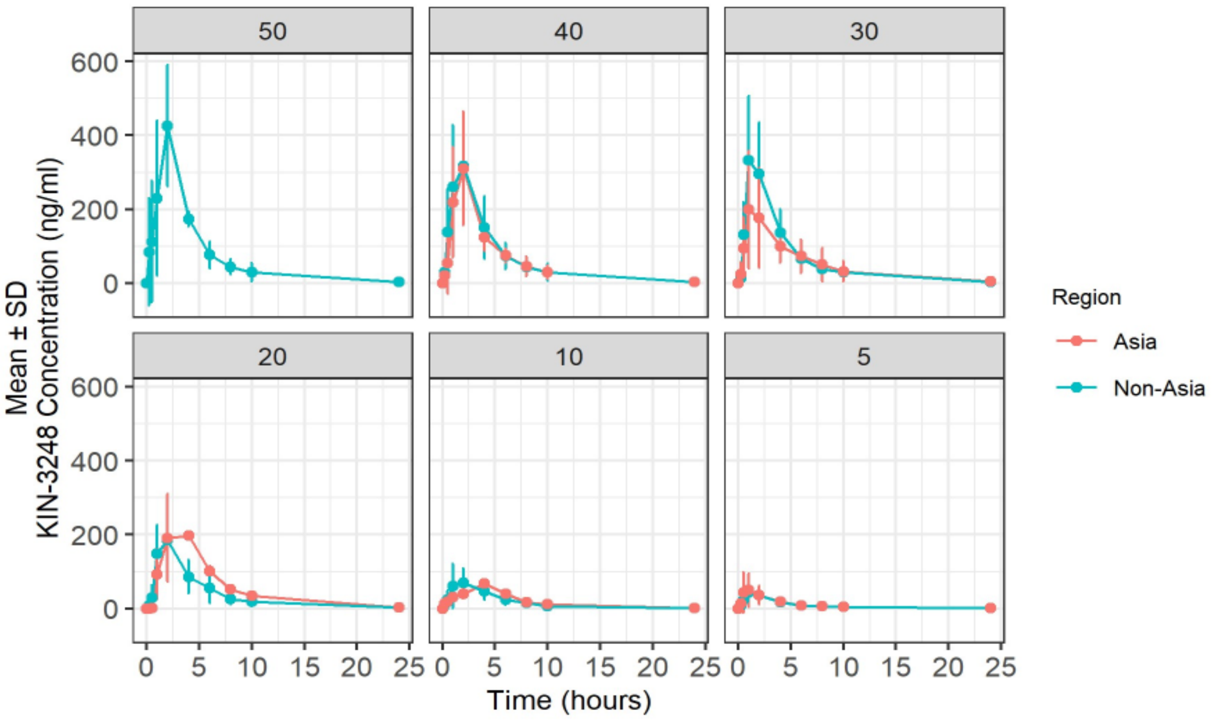

Supplement: Supplementary Figure 2 — Supplemental Figure 2 - Mean KIN-3248 Concentrations over time for Asian versus non-Asian Patients [file crc-24-0137-s03.pdf]
