# Supplementary material for: A Phase I Study of KIN-3248, an Irreversible Small-molecule Pan-FGFR Inhibitor, in Patients with Advanced FGFR2/3-driven Solid Tumors
Source: Cancer Res Commun. 2024 Apr 30;4(4):1165–73. doi: 10.1158/2767-9764.CRC-24-0137 (PMC11060137; doi:10.1158/2767-9764.CRC-24-0137)

Supplemental Figure 3: Serum Phosphate Over Time by KIN-3248 Dose Level. Locally tested serum phosphate levels (mg/dL) from each patient (open circles with solid color lines) plotted over time (weeks relative to C1D1; screening through 6 weeks on study). Dotted line at zero on x-axis marks C1D1. Thin, horizontal dashed lines represent the Lab Low (blue) and Lab High (red) values from the various local labs where samples were tested. Thick black lines show the smoothed conditional means (loess trend line with confidence interval) for all patients at each dose level. For all dose levels, serum phosphate elevations occur within the first week of treatment. Initial phosphate elevations are dose dependent. Management of hyperphosphatemia likely explains the gradual reductions in serum phosphate after 2 weeks (See Table 5 of KN-4802 protocol). Protocol Hyperphosphatemia References: Grade 2 = 5.6-6.9 mg/dL, Grade 3 = 7.0-9.9 mg/dL.

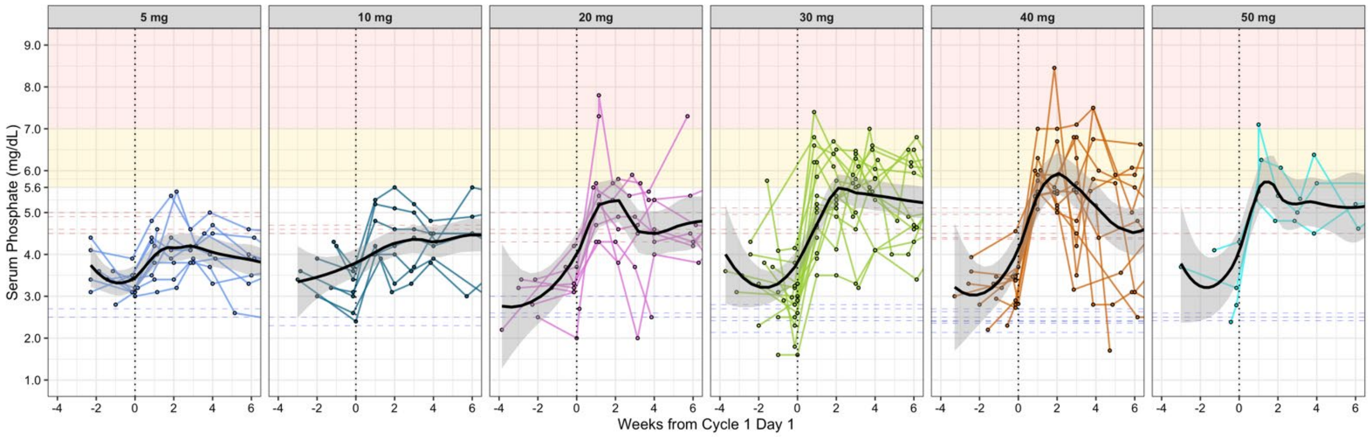

Supplement: Supplementary Figure 3 — Supplemental Figure 3 - Serum Phosphate Over Time by KIN-3248 Dose Level [file crc-24-0137-s04.pdf]
