# Supplementary material for: A Phase I Study of KIN-3248, an Irreversible Small-molecule Pan-FGFR Inhibitor, in Patients with Advanced FGFR2/3-driven Solid Tumors
Source: Cancer Res Commun. 2024 Apr 30;4(4):1165–73. doi: 10.1158/2767-9764.CRC-24-0137 (PMC11060137; doi:10.1158/2767-9764.CRC-24-0137)

Supplemental Figure 4: Pharmacodynamic effect of KIN-3248 treatment was evaluated in serial sections of paired biopsy samples by measuring changes in phospho-ERK and DUSP6 gene expression. Total ERK was also measured and used to calculated the ratio of phosphor-ERK to total ERK (pERK_ratio). H-Scores from immunohistochemistry, ratios, and RNAscope scores are presented for each patient, biopsy, and analyte as box and whisker plots (A). A summary of percent change ((ScoreOn-Treatment-ScoreScreening)/ ScoreScreening*100) of each marker presented as box and whisker plots for each dose level (B) or across all cohorts (C).

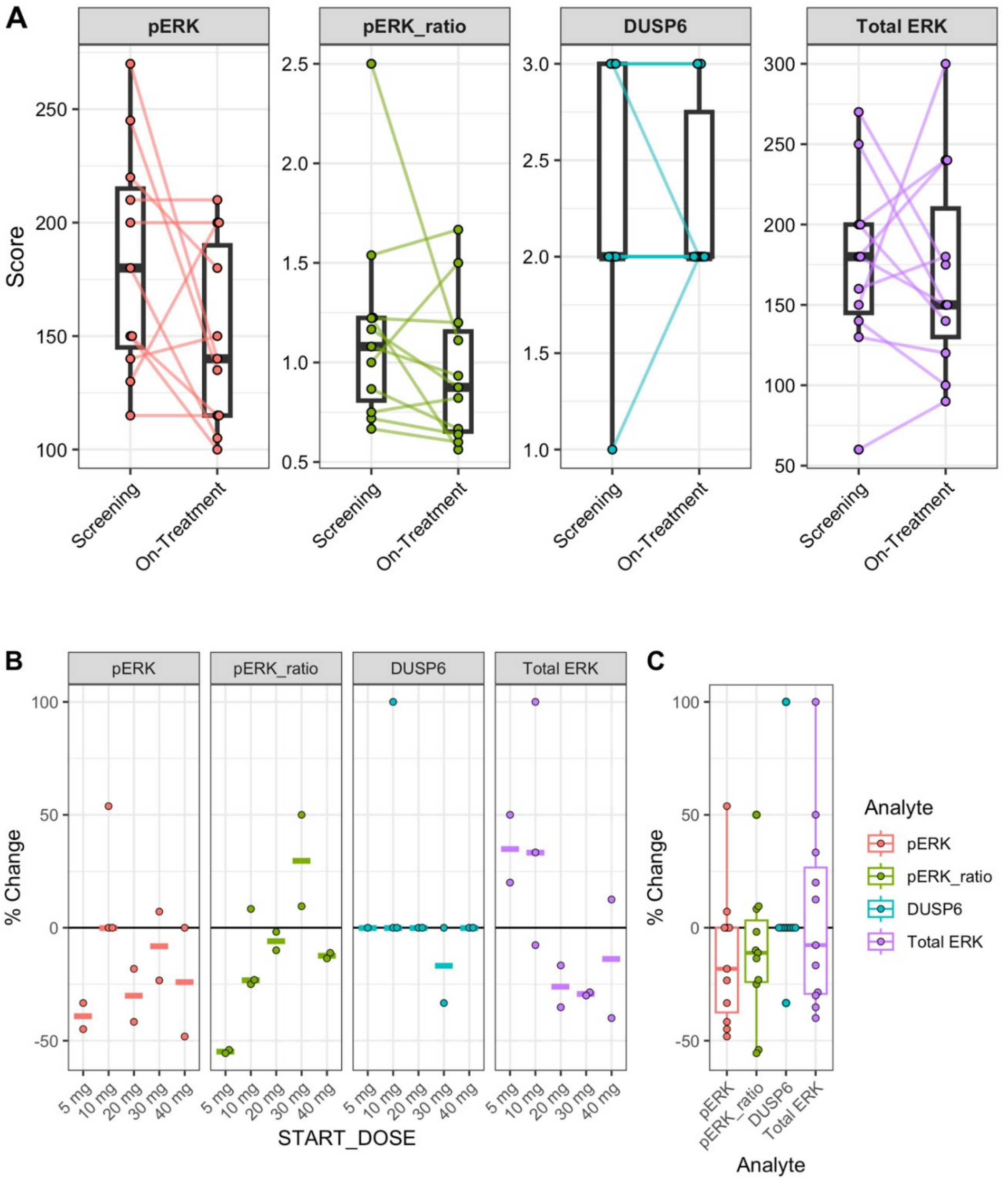

Supplement: Supplementary Figure 4 — Supplemental Figure 4 - Pharmacodynamic effect of KIN-3248 treatment was evaluated in serial sections of paired biopsy samples by measuring changes in phospho-ERK and DUSP6 gene expression. [file crc-24-0137-s05.pdf]
