# Supplementary material for: A Phase I Study of KIN-3248, an Irreversible Small-molecule Pan-FGFR Inhibitor, in Patients with Advanced FGFR2/3-driven Solid Tumors
Source: Cancer Res Commun. 2024 Apr 30;4(4):1165–73. doi: 10.1158/2767-9764.CRC-24-0137 (PMC11060137; doi:10.1158/2767-9764.CRC-24-0137)

Supplemental Figure 5: Somatic Mutation Profiles in Baseline ctDNA. Somatic mutations identified from ctDNA are presented descriptively as an Oncoplot using maftools package (32) from bioconductor. The oncoplot includes all genes that were altered in ≥ 4 patients or actionable oncogenes (OncoKB Level 1) with oncogenic mutations in ≥ 1 patient. The TMB stacked barplot shows the number of somatic and non-silent mutations identified in a sample. Amp: ctDNA amplification ≥ 2.6. Amplifications are not counted in the stacked TMB bars on top of the oncoplot. FGFR_Status indicates whether the FGFR2 or FGFR3 alteration identified locally was confirmed by the baseline ctDNA analysis.

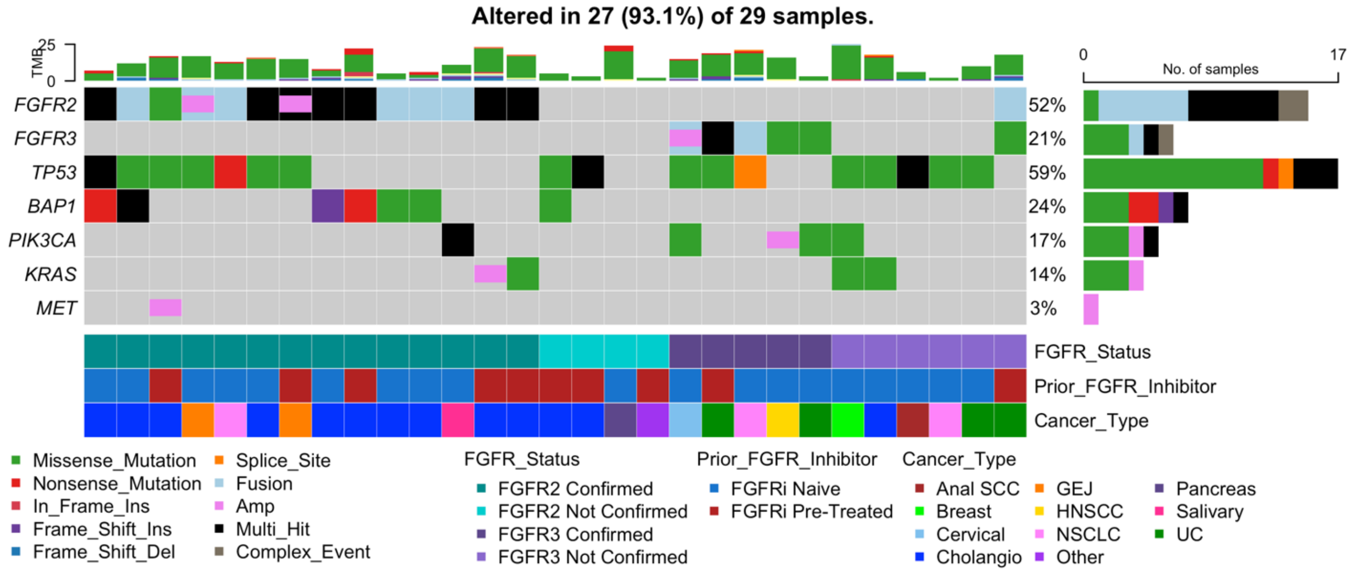

Supplement: Supplementary Figure 5 — Supplemental Figure 5 - Somatic Mutation Profiles in Baseline ctDNA [file crc-24-0137-s06.pdf]
