# Supplementary material for: A Phase I Study of KIN-3248, an Irreversible Small-molecule Pan-FGFR Inhibitor, in Patients with Advanced FGFR2/3-driven Solid Tumors
Source: Cancer Res Commun. 2024 Apr 30;4(4):1165–73. doi: 10.1158/2767-9764.CRC-24-0137 (PMC11060137; doi:10.1158/2767-9764.CRC-24-0137)
Supplement: Supplementary Table 1 — Supplemental Table 1 - Pathogenic / Likely-pathogenic FGFR2 Gene Alterations [file crc-24-0137-s07.pdf]

Supplemental Table 1: Pathogenic / Likely-pathogenic FGFR2 Gene Alterations

Alteration Type	Functional Description	FGFR2 Alteration		
Fusion	Activating via upregulation and / or ligand-independent dimerization	FGFR2-ACLY	FGFR2-DNAJC12	FGFR2-NOL4
		FGFR2-AFF3	FGFR2-DSP	FGFR2-NRAP
		FGFR2-AFF4	FGFR2-EEA1	FGFR2-NRBF2
		FGFR2-AHCYL1	FGFR2-EIF4ENIF1	FGFR2-OFD1
		FGFR2-ARHGAP22	FGFR2-ERC1	FGFR2-OPTN
		FGFR2-ARHGAP24	FGFR2-FAM160B1	FGFR2-PAH
		FGFR2-ATAD2	FGFR2-FAM67A	FGFR2-PAWR
		FGFR2-ATF2	FGFR2-FILIP1	FGFR2-PIBF1
		FGFR2-BICC1	FGFR2-GAB2	FGFR2-POC1B
		FGFR2-BICD1	FGFR2-GOPC	FGFR2-POF1B
		FGFR2-CASP7	FGFR2-INSC	FGFR2-PPHLN1
		FGFR2-CCDC158	FGFR2-KCTD1	FGFR2-PXN
		FGFR2-CCDC170	FGFR2-KIAA1217	FGFR2-RABGAP1L
		FGFR2-CCDC6	FGFR2-KIAA1598	FGFR2-RASSF4
		FGFR2-CDC42BPB	FGFR2-KIAA1967	FGFR2-ROBO2
		FGFR2-CEP128	FGFR2-MACF1	FGFR2-RPAP3
		FGFR2-CIT	FGFR2-MATR3	FGFR2-SFI1
		FGFR2-COL16A1	FGFR2-MCU	FGFR2-SHROOM3
		FGFR2-CTNNA3	FGFR2-MGEA5	FGFR2-SLMAP
		FGFR2-DBP	FGFR2-NEDD4L	FGFR2-SOGA1
		FGFR2-SORBS1	FGFR2-TBC1D1	FGFR2-USP10
		FGFR2-SPERT	FGFR2-TCTN3	FGFR2-VCL
		FGFR2-SPICE1	FGFR2-TFEC	FGFR2-WAC
		FGFR2-STRN4	FGFR2-TRIM8	FGFR2-WDHD1
		FGFR2-TACC1	FGFR2-TTC28	FGFR2-ZMYM4
		FGFR2-TACC2	FGFR2-TXLNB	FGFR2-ZNF521
		FGFR2-TACC3	FGFR2-USH2A	

Alteration Type	Functional Description	FGFR2 Alteration		
Insertion-Deletion	Extracellular domain activating mutation	H167_N173del	P170_K176del	P256_G261delins R
		P263_A266del	T268_D273delinsS	V280_K292del
		P286_K292del	I288_E295delinsT	W290_I291delinsC
Missense	Activating Loop	K659M	K659N	
	Extracellular domain activating mutation	D101Y	P253R	T341P
		R203C	F276C	S372C
		R251Q	W290R	Y375C
		S252W		
	Gatekeeper	V564F	V564I	V564L
	Molecular Brake / Regulatory Triad	N549H	N549T	K641N
		N549K	E565G	K641R
		N549S	E565A	
	Other kinase domain activating mutations	K526E	V562L	R678G
		M535I	L617F	H682L
		M537I	L617M	K714R
		M538I	L617V	E731K
		I547V		
	Transmembrane domain activating mutation	Y381D	C382R	M391R
Duplication	Extracellular domain activating mutation	S267_D273dup		

Note: Amino acid positions based on RefSeq NM_000141.

Pathogenic / Likely-pathogenic FGFR3 Gene Alterations

Alteration Type	Functional Description	FGFR3 Alteration		
Fusion	Activating via upregulation and / or ligand-independent dimerization	FGFR3-TACC3	FGFR3-BAIAP2L1	IGH-FGFR3
Missense	Extracellular domain activating mutation	R248C	G370C	Y373C
		S249C	S371C	G375C
	Gatekeeper	V555M		
	Molecular Brake	N540K	N540S	
	Activating Loop	K650E	K650R	K650N
		K650Q	K650M	K650T
		M528I	D641G	Y647C

Alteration Type	Functional Description	FGFR3 Alteration		
	Other kinase domain activating mutations	I538V	D641N	R669G
	Transmembrane domain activating mutation	G380R	G382D	A391E

Note: Amino acid positions based on RefSeq NM_000142.
