# Supplementary material for: A Phase I Study of KIN-3248, an Irreversible Small-molecule Pan-FGFR Inhibitor, in Patients with Advanced FGFR2/3-driven Solid Tumors
Source: Cancer Res Commun. 2024 Apr 30;4(4):1165–73. doi: 10.1158/2767-9764.CRC-24-0137 (PMC11060137; doi:10.1158/2767-9764.CRC-24-0137)
Supplement: Supplementary Table 2 — Supplemental Table 2 - Management of Hyperphosphatemia Observed with FGFR Inhibitors [file crc-24-0137-s08.pdf]

Supplemental Table 2: Management of Hyperphosphatemia Observed with FGFR Inhibitors

Phosphorus Level	Grade	Investigator Guidance for Management
–>ULN and ≤ 5.5 mg/dL (1.7 mmol/L)	1	• Continue KIN-3248 treatment<br>• Reinforce low-phosphate diet
5.6 – 6.9 mg/dL (1.8 – 2.3 mmol/L)	2	• Continue KIN-3248 treatment at current dose with low-phosphate diet<br>• Start phosphate binder at lowest possible dose; recheck phosphorous levels in 1 week<br>• Dose escalation of phosphate binder or addition of phosphaturic agent such as acetazolamide should be considered if phosphorous levels remain elevated after 7 days of initial intervention.
7.0 – 9.9 mg/dL (2.3 – 3.1 mmol/L)	3	• No interruption in KIN-3248 treatment; reduce dose to the next lower dose.<br>• Maximum recommended dose of phosphate binder in combination with phosphaturic agent such as acetazolamide 250 mg twice or three times a day<br>• Recheck phosphorous levels weekly for 2 weeks<br>• If serum phosphate resolves to < 7 mg/dL (<2.3 mmol/L), continue at the reduced dose.<br>• If serum phosphate has not resolved to < 7 mg/dL (<2.3 mmol/L) within 14 days, reduce the dose further from the previous reduced dose.<br>• If serum phosphate still has not resolved to < 7 mg/dL (<2.3 mmol/L) after 14 days of the 2nd dose reduction, interrupt dosing until it is resolved to 7 mg/dL (2.3 mmol/L) before resuming dosing at the reduced dose prior to dose interruption.
≥ 10.0 mg/dL (≥ 3.2 mmol/L)	4	• Interrupt KIN-3248 treatment<br>• Maximum recommended dose of phosphate binder in combination with phosphaturic agent such as acetazolamide 250 mg twice or three times a day. Recheck levels twice per week until < 7 mg/dL (< 2.3 mmol/L) then they may restart KIN-3248 at a reduced dose level.<br>• Permanent discontinuation of KIN-3248 if phosphorus levels > 7 mg/dL (>2.3 mmol/L) after 14 days.
