# Supplementary material for: A Phase I Study of KIN-3248, an Irreversible Small-molecule Pan-FGFR Inhibitor, in Patients with Advanced FGFR2/3-driven Solid Tumors
Source: Cancer Res Commun. 2024 Apr 30;4(4):1165–73. doi: 10.1158/2767-9764.CRC-24-0137 (PMC11060137; doi:10.1158/2767-9764.CRC-24-0137)
Supplement: Supplementary Table 3 — Supplemental Table 3 - Summary of Pharmacokinetic Parameters of KIN-3248 (Cycle 1 Day 1) [file crc-24-0137-s09.pdf]

Supplemental Table 3: Summary of Pharmacokinetic Parameters of KIN-3248 (Cycle 1 Day 1)

Dose (mg)	n	C_{max} (ng/mL)	$C_{max\ p}$ (ng/mL/ mg)	T_{max} (h)	AUC_{0-24} (h*ng/ml)	AUC_{0-24_D} (h*ng/ml/ mg)	C_{min} (ng/ml)	$t_{1/2}$ (h)
Geo Mean (Geo CV%)								
5	9	45.9 (57.8%)	9.18 (57.8%)	1.00 (0.500-4.00)	186 (25.8%)	37.2 (25.8%)	0.825 (35.9%) #	4.53 (65.1%)
10	7	76.4 (46.5%)	7.64 (46.5%)	4.00 (1.00-4.00)	381 (29.5%)	38.1 (29.5%)	1.29 (47.4%) †	3.96 (40.7%)
20	8	187 (32.5%)	9.35 (32.5%)	2.00 (1.00-4.00)	845 (48%)	42.2 (48%)	2.03 (92.6%) †	4.03 (27.1%)
30	16	268 (63.4%)	8.92 (63.4%)	1.50 (1.00-4.00)	1180 (31.8%) †	39.2 (31.8%) †	3.28 (110.6%) †	4.53 (42.4%)
40	11	285 (60.5%)	7.12 (60.5%)	2.00 (1.00-2.00)	1260 (42.2%)	31.5 (42.2%)	3.63 (57.3%) ##	4.53 (42.4%)
