# Supplementary material for: A Phase I Study of KIN-3248, an Irreversible Small-molecule Pan-FGFR Inhibitor, in Patients with Advanced FGFR2/3-driven Solid Tumors
Source: Cancer Res Commun. 2024 Apr 30;4(4):1165–73. doi: 10.1158/2767-9764.CRC-24-0137 (PMC11060137; doi:10.1158/2767-9764.CRC-24-0137)
Supplement: Supplementary Table 4 — Supplemental Table 4 - FGFRi pre-treated patients with kinase domain resistance mutations identified in centrally tested ctDNA [file crc-24-0137-s10.pdf]

Supplemental Table 1: **FGFRi pre-treated patients with kinase domain resistance mutations identified in centrally tested ctDNA**

**Tumor Type**	**Cholangiocarcinoma**	**Cholangiocarcinoma**	**Cholangiocarcinoma**	**Gastric**
Primary Driver	FGFR2-BICC1 Fusion	FGFR2-INA Fusion	FGFR2-BICC1 Fusion	FGFR2-CTNNB1 Fusion
Resistance	FGFR2 p.E565K<br>FGFR2 p.V564F<br>FGFR2 p.E565G<br>FGFR2 p.N549H<br>FGFR2 p.V564I<br>FGFR2 p.T635I<br>FGFR2 p.K641R	FGFR2 p.N549K<br>FGFR2 p.V564F<br>FGFR2 p.M537_M538delinsIL<br>FGFR2 p.N549H<br>FGFR2 p.M537I	FGFR2 p.L617F (c.1851G>T)<br>FGFR2 p.L617F (c.1851G>C)<br>FGFR2 p.V564I<br>FGFR2 p.M537I (c.1611G>T)<br>FGFR2 p.M537I (c.1611G>A)<br>FGFR2 p.M537I (c.1611G>C)<br>FGFR2 p.N549K (c.1647T>A)<br>FGFR2 p.N549K (c.1647T>G)<br>FGFR2 p.I548N	FGFR2 p.L617M<br>FGFR2 p.L617F
